# Modulation of the hippo-YAP pathway by cyclic stretch in rat type 2 alveolar epithelial cells—a proof-of-concept study

**DOI:** 10.3389/fphys.2023.1253810

**Published:** 2023-10-09

**Authors:** Xi Ran, Sabine Müller, Coy Brunssen, Robert Huhle, Martin Scharffenberg, Christian Schnabel, Thea Koch, Marcelo Gama de Abreu, Henning Morawietz, Jorge M. C. Ferreira, Jakob Wittenstein

**Affiliations:** ^1^ Department of Intensive Care Medicine, Chongqing General Hospital, Changqing, China; ^2^ Department of Anesthesiology and Intensive Care Medicine, Pulmonary Engineering Group, University Hospital Carl Gustav Carus Dresden, TUD Dresden University of Technology, Dresden, Germany; ^3^ Division of Vascular Endothelium and Microcirculation, Department of Medicine III, University Hospital and Medical Faculty Carl Gustav Carus, TUD Dresden University of Technology, Dresden, Germany; ^4^ Department of Anesthesiology and Intensive Care Medicine, Clinical Sensoring and Monitoring Group, University Hospital Carl Gustav Carus Dresden, TUD Dresden University of Technology, Dresden, Germany; ^5^ Department of Intensive Care and Resuscitation, Anesthesiology Institute, Cleveland Clinic, Cleveland, OH, United States; ^6^ Department of Outcomes Research, Anesthesiology Institute, Cleveland Clinic, Cleveland, OH, United States

**Keywords:** alveolar epithelial cells, cyclic stretch, VILI, cytokines, hippo-YAP pathway, inflammation

## Abstract

**Background:** Mechanical ventilation (MV) is a life supporting therapy but may also cause lung damage. This phenomenon is known as ventilator-induced lung injury (VILI). A potential pathomechanisms of ventilator-induced lung injury may be the stretch-induced production and release of cytokines and pro-inflammatory molecules from the alveolar epithelium. Yes-associated protein (YAP) might be regulated by mechanical forces and involved in the inflammation cascade. However, its role in stretch-induced damage of alveolar cells remains poorly understood. In this study, we explored the role of YAP in the response of alveolar epithelial type II cells (AEC II) to elevated cyclic stretch *in vitro*. We hypothesize that Yes-associated protein activates its downstream targets and regulates the interleukin-6 (IL-6) expression in response to 30% cyclic stretch in AEC II.

**Methods:** The rat lung L2 cell line was exposed to 30% cyclic equibiaxial stretch for 1 or 4 h. Non-stretched conditions served as controls. The cytoskeleton remodeling and cell junction integrity were evaluated by F-actin and Pan-cadherin immunofluorescence, respectively. The gene expression and protein levels of IL-6, Yes-associated protein, Cysteine-rich angiogenic inducer 61 (Cyr61/CCN1), and connective tissue growth factor (CTGF/CCN2) were studied by real-time polymerase chain reaction (RT-qPCR) and Western blot, respectively. Verteporfin (VP) was used to inhibit Yes-associated protein activation. The effects of 30% cyclic stretch were assessed by two-way ANOVA. Statistical significance as accepted at *p* < 0.05.

**Results:** Cyclic stretch of 30% induced YAP nuclear accumulation, activated the transcription of Yes-associated protein downstream targets Cyr61/CCN1 and CTGF/CCN2 and elevated IL-6 expression in AEC II after 1 hour, compared to static control. VP (2 µM) inhibited Yes-associated protein activation in response to 30% cyclic stretch and reduced IL-6 protein levels.

**Conclusion:** In rat lung L2 AEC II, 30% cyclic stretch activated YAP, and its downstream targets Cyr61/CCN1 and CTGF/CCN2 and proinflammatory IL-6 expression. Target activation was blocked by a Yes-associated protein inhibitor. This novel YAP-dependent pathway could be involved in stretch-induced damage of alveolar cells.

## 1 Introduction

Mechanical ventilation (MV) is a life-saving therapy that aims to maintain adequate gas exchange and to reduce the work of breathing in intensive care patients. However, MV can worsen or even initiate lung injury, known as ventilator-induced lung injury (VILI) (Beitler et al., 2016; Botta et al., 2021). *In vivo* and *in vitro* studies have shown that excessive stretch during MV can promote the production and release of cytokines and pro-inflammatory molecules, such as Interleukin-6 (IL-6), ultimately leading to biotrauma (Kobayashi et al., 2012; Michalick et al., 2017; Grune et al., 2019). Despite decades of intensive research, there is currently no causal therapy of stretch-induced pulmonary cell damage. Therefore, it is critical to further explore the pathogenesis and search for potential therapeutic targets to minimize stretch-induced biotrauma.

Alveolar epithelial cells type II (AEC II), one of the main types of the alveolar epithelium, are located in the alveolar wall surface in contact to the atmosphere. They are exposed to breathing-related mechanical loading. AEC II are essential to maintain alveolar integrity and normal lung function by secreting the surfactant proteins and differentiating as progenitor cells into AEC type I cells after lung injury (Herzog et al., 2008). AEC II also produces cytokines affecting immune cells (Crosby et al., 2011; Umannová et al., 2011). Furthermore, the epithelium is involved in cellular response to VILI (Suki and Hubmayr, 2014; Tanaka et al., 2017). In a previous study we could show that excessive mechanical loading enhanced the pro-inflammatory mRNA expression of pro-inflammatory molecules in AEC II *in vitro* (Ferreira et al., 2022). However, the molecular mechanisms underlying this process remain unclear. In particular, the early cellular events occurring in response to cyclic stretch are not well-understood. In vascular smooth muscle cells, we found an induction of immediate-early genes in response to short-term cyclic stretch (Morawietz et al., 1999). Another mechanism activated in response to cyclic stretch might be the Hippo-YAP pathway (Dupont et al., 2011; Aragona et al., 2013; Elbediwy and Thompson, 2018).

The Yes-associated protein (YAP) is a transcriptional coactivator and serves as a nuclear relay of mechanical signals (Dupont et al., 2011). It interacts with transcriptional enhanced associate domain (TEAD) family member transcription factors, induces the transcription of Cysteine-rich angiogenic inducer 61 (Cyr61/CCN1) and connective tissue growth factor (CTGF/CCN2) as well as promotes cell growth, proliferation, and survival. YAP and Transcriptional coactivator with PDZ-binding motif (TAZ) can be involved in the initial cellular responses to mechanical forces like shear stress or cyclic strain under physiological and pathophysiological conditions and translate them into cell-specific transcriptional programs (Codelia et al., 2014; Panciera et al., 2017). In addition, inhibition of YAP can diminish inflammation in response to disturbed flow (Taniguchi et al., 2015; Li et al., 2019). Thus, YAP may serve as a mechanosensitive transducer triggering the inflammatory cascade in mechanical stretch-induced lung injury. Yet, this concept has not been investigated.

This study aimed to explore the role of YAP in the regulation of the inflammatory response in response to high-level cyclic stretch in AEC II *in vitro*. We hypothesized that YAP activation modulates the upregulation of IL-6 in response to a 30% cyclic stretch in AEC II. Thus, a L2 cell line was exposed to a 30% cyclic stretch over 1–4 h and assessed the expression and activation of YAP and IL-6. In addition, we also investigated the effects of YAP inhibition on the IL-6 expression. The results of this study provide novel insights into the molecular mechanisms of cyclic stretch-induced alveolar damage and VILI–dependent development of biotrauma. This may identify novel targets for potential therapeutic strategies.

## 2 Materials and methods

### 2.1 Cell culture

Rat AEC Type II (ATCC, L2 cell line # CCL-149™) were used. L2 cells at passage 7 were seeded onto six-well BioFlex Collagen I coated plates (Flexcell International Corporation, Burlington, NC, United States) at a density of 1.5 × 10^5^ cells/well in Dulbecco’s modified Eagle medium (DMEM, with High Glucose, L-glutamine, Phenol Red and Sodium Pyruvate, Gibco, # 11995073) supplemented with 10% fetal bovine serum (FBS, Gibco) and 50 μg/mL gentamycin sulfate (Gibco, # 15710049). Cells were incubated at 37°C, 21% O2 and 6.5% CO_2_ for 24 h. Sixteen hours before stretch experiments, cells were washed twice with sterile phosphate-buffered saline (PBS) and then incubated with DMEM (without Phenol Red, Merck Sigma-Aldrich, #D1145) containing 1% FBS, 50 µg gentamycin sulfate/mL, 4 mM L-alanyl-L-glutamine (Merck Sigma-Aldrich, # G8541), and 1mM Sodium Pyruvate (Gibco, # 11360070).

### 2.2 YAP inhibitor

Verteporfin (Merck Sigma-Aldrich, SML0534) stock solution of 1 M was prepared in dimethyl sulfoxide (DMSO) according to the manufacturer’s instructions. One hour prior to the YAP inhibitor experiment, cells were pretreated with 2 µM verteporfin. An equal volume of DMSO was added to control cells.

### 2.3 Cyclic stretch

We used a custom-made stretching device for cell cyclic-stretching, as previously described (Rentzsch et al., 2017; Ferreira et al., 2022) Briefly, the device utilizes three cylindrical intenders powered by a stepper motor (vertical motion, Maxon Motor AG, Sachseln, Switzerland) to apply a homogeneous cyclic equibiaxial stretch on three wells of a BioFlex plate with silicone membrane, while the other three wells served as non-stretched controls. The driving motor and stretching parameters were controlled by a custom-made program (LabView, National Instruments, Austix, TX, United States). The stretch group was subjected to a magnitude of 30% strain at 0.5 Hz frequency with a stretching/relaxation ratio of 1:1 (sinusoidal pattern) for 1 or 4 h, as previously describted (Ferreira et al., 2022).

### 2.4 RNA isolation and quantification

Total RNA was extracted with the NucleoSpin RNA Plus, Mini kit (Macherey-Nagel, Düren, Germany, # 740984.50) according to the manufacturer’s protocol. RNA concentrations were evaluated using a spectrophotometer, and the purity was assessed based on the ratio of absorbance at 260 nm and 280 nm (A260–A280). Subsequently, RNA was reversely transcribed using the qScript Ultra SuperMix (Quantabio, Beverly, MA, United States, #95217-100) and the resulting cDNA was used for real-time polymerase chain reaction (PCR) with the PerfeCTa SYBR Green FastMix (Quantabio, Beverly, MA, United States, # 95071). The gene expression of IL-6, Cyr61/CCN1, and CTGF/CCN2 was quantified by real-time PCR on the PCR MyiQ™ 2 Cycler (Biorad, Kabelsketal, Germany), using the IQ 5 software (version: 2.1.97.1001) and the following conditions: 95°C for 15 s, followed by 45 cycles at 95°C for 5 s, 60°C for 15 s, and extension at 68°C for 10 s. The primers (Eurofins Genomics, Ebersberg, Germany) are listed in Table 1. Cycle threshold (Ct) values of target genes were normalized to the housekeeping gene β-Actin.

**TABLE 1:** Primers for real time PCR.

Gene	Primer	Sequence (5′-3′)
Cyr61/CCN1	Forward	GTG CCG CCT GGT GAA AGA GA
Cyr61/CCN1	Reverse	GCT GCA TTT CTT GCC CTT TTT TAG
CTGF/CCN2	Forward	ACC TGT GCC TGC CAT TAC AA
CTGF/CCN2	Reverse	CTC ACT TCG GTG GGG TGT TT
β-Actin	Forward	AAC CCT AAG GCC AAC CGT GAA A
β-Actin	Reverse	AGT CCA TCA CAA TGC CAG TGG T
IL-6	Forward	GAC AAA GCC AGA GTC ATT CAG AG
IL-6	Reverse	TTG GAT GGT CTT GGT CCT TAG CC

### 2.5 Cell fractionation

Cells were washed once with ice-cold PBS supplemented with 1 × protease inhibitor cocktail (Roche Applied Science, # 11836153001) and incubated in the hypotonic buffer containing 10 mM 4-(2-hydroxyethyl)-1-piperazineethanesulfonic acid (HEPES), 10 mM KCl, 1.5 mM MgCl_2_, and 1 mM DL-Dithiothreitol (DTT) on ice for 10 min. Cells were lyzed by passaging 10 times through a 27G needle attached to a1-mL syringe pipetted for 40 s, 14000 x g, 4°C. Supernatants cytosolic protein were collected. The nuclear pellet was resuspended and homogenized in buffer (pH 7.5) containing 50 mM Tris–HCl buffer, 150 mM sodium chloride, 2 mM Ethylenediaminetetraacetic acid (EDTA), 1% Triton X-100, supplemented with 1 × protease inhibitor cocktail (Roche Applied Science, # 11836153001) and incubated on ice for 30 min. Subsequently, insoluble proteins were harvested from the nuclear extract by high-speed centrifugation (20 min, 14,000 × g, 4°C).

### 2.6 Protein extraction and Western blot

Cells were homogenized in lysis buffer (pH 7.5) containing 50 mM Tris–HCl, 150 mM sodium chloride, 2 mM Ethylenediaminetetraacetic acid (EDTA), 1% Triton X-100, supplemented with 1 × protease inhibitor cocktail (Roche Applied Science, # 11836153001). The denatured proteins (10 μg each well) were separated on a sodium dodecyl sulfate–polyacrylamide gel (4%–20% Mini-PROTEAN TGX Gels, Bio-Rad Laboratories, Hercules, CA, United States, #4561094) and transferred to polyvinylidene fluoride membrane (0.2 µm, Bio-Rad, #1704272) using Semi-Dry blotting system (TransBlot^®^ Turbo™ Transfer System, Bio-Rad). The membranes were blocked in Tris-buffered saline containing 0.1% Tween 20% and 5% low fat milk, and then incubated with the following primary antibodies overnight at 4°C: anti-IL-6 (1:500, Santa Cruz Biotech, Inc., Dallas, TX, United States, #sc-57315), anti-GAPDH (1:20,000, Calbiochem, San Diego, CA, United States, #CB1001), anti-YAP (1:2,000, Cell Signaling Technology, Danvers, MA, United States, #14074), anti-Phospho-YAP Ser127 (pYAP, 1:2,000, Cell Signaling Technology, #13008), anti-Lamin A/C (1:1,000, Cell signaling, #4777T), anti-α-Tubulin (1:1,000, Thermo Fisher Scientific, Waltham, MA, United States, #A11126), anti-Cyr61/CCN1 (1:1,500, Proteintech, Rosemont, IL, United States, #26689-1-AP), anti-CCN2/CTGF (1:700, Santa Cruz, #Sc14939), anti-Vinculin (1:500, Bio-Rad AbD Serotec, Neuried, Germany, #B1017), andβ-Actin (1:20,000, Merck Sigma-Aldrich,# A3854). After washing, membranes were incubated with secondary antibodies anti-mouse (GE Healthcare, #381334, at a dilution of 1:1,000 for IL-6, Lamin A/C, and α-Tubulin, a dilution of 1:4,000 for GAPDH), or goat anti-rabbit (BioRad, #1721019, at a dilution of 1:1,000 for Cyr61/CCN1, 1:2,000 for YAP and pYAP), or mouse anti-goat (Jackson ImmunoResearch, #205-035-108, at a dilution of 1:1,000 for CTGF/CCN2) at room temperature for 1 h. The blots were developed with WesternBright Chemiluminescence Substrate Quantum (Advansta, San Jose, CA, United States, #541013) and visualized employing a Fujifilm LAS-3000 Luminescent Image Analyzer (Fuji Film, Minato, Japan, 2006). Densitometric analysis of the protein bands was performed using the ImageJ software (Schneider et al., 2012).

### 2.7 Immunofluorescence

To examine the effects of 30% cyclic stretch on cytoskeletal remodeling, stress fibers and intercellular junctions were visualized by immunofluorescence of F-actin (filamentous actin) and Pan-cadherin (all members of the cadherin family of proteins including N-cadherin, E-cadherin, P-cadherin, and R-cadherin). Briefly, cells were cultured in six Flexcell plates (Flexcell International Corporation, Burlington, NC, United States) for 48 h until they reached confluence. Cells were washed twice with ice-cold PBS and fixed with 4% paraformaldehyde (PFA) on ice for 5 min. The PFA was washed away twice with PBS. Subsequent permeabilization was performed by incubating cells with 0.1% Triton X-100 in PBS at room temperature for 5 min. Afterwards, cells were blocked with 3% bovine serum albumin in PBS at room temperature for 1 h, followed by overnight incubation with Invitrogen™ Alexa Fluor™ 488 Phalloidin (1:500, Molecular Probes, Inc., Eugene, OR, United States, # A12379) or primary antibodies against YAP (1:200, Cell Signaling Technology, #14074), anti-Surfactant Protein C (1:200, Merck Millipore Corporation, Burlington, MA, United States, #AB3786), and Pan-cadherin (1:300, Merck Sigma-Aldrich, C1821) at 4°C. After washing with PBS, cells were incubated with secondary antibodies including Alexa Fluor 488 goat anti-mouse IgG (1:1,000, Thermo Fisher Scientific, Waltham, MA, United States, # A-11034) and Alexa Fluor 594 goat anti-mouse IgG (1:200, Thermo Fisher Scientific, # A-11005) at room temperature for 1 h, as well as 4 h, 4’,6-diamidino-2-phenylindole (DAPI, 1:200, Merck Sigma-Aldrich, # D-9542) at room temperature for 30 min. The stained cells attached to the silicone membranes were cut off the plates and mounted inverted on slides using the anti-fade mounting reagent (MOWIOL, Calbiochem, # 475904).

Immunofluorescence images were acquired with a Zeiss LSM880 confocal laser scanning microscope with Airyscan using a ×40 oil immersion objective at the Cellular Imaging Facility of the Medical Theoretical Centre, Faculty of Medicine of the TUD Dresden University of Technology. Fluorescence was detected at the wavelengths: 594 nm (Alexa 594), 488 nm (Alexa 488), and 405 nm (DAPI). Image processing was performed by ImageJ (Schneider et al., 2012).

### 2.8 Statistical analysis

Data are reported as median ± interquartile range (IQR). Normal distribution of data was tested by the Shapiro-Wilk test. Comparisons among groups were conducted with Kruskal–Wallis test. The data were analyzed using IBM SPSS Statistics (Version 26) (IBM Corp, 2017). Statistical significance was accepted at *p* < 0.05.

## 3 Results

### 3.1 Effects of cyclic stretch on cytoskeletal remodeling and membrane deformation in cultured L2 cells

**FIGURE 1:**
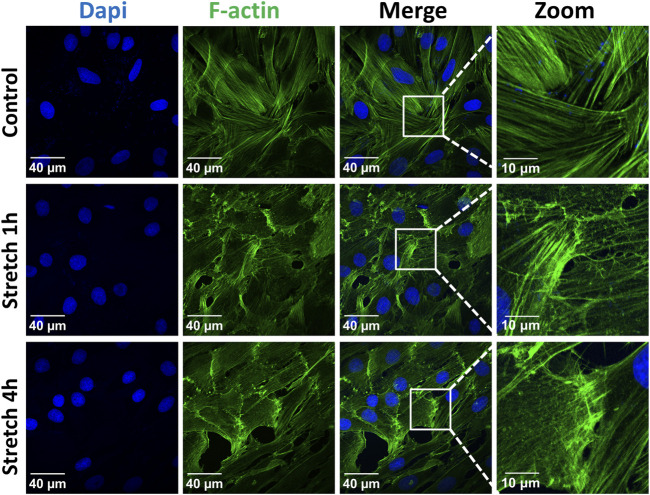
Effect of 30% cyclic stretch on cytoskeletal remodeling in rat alveolar epithelial type II L2 cells. Cells were grown on silicone membranes and subjected to 30% cyclic stretch for 1–4 h. Cells kept under static conditions (non-stretched) were used as the control. Cytoskeletal remodeling was visualized by immunostaining of F-actin (filamentous actin). Labelling of cell nucleus with DAPI (blue) and of F-actin with phalloidin (green). Scale bar: 40 μm; 10 µm (zoom). n = 6.

**FIGURE 2:**
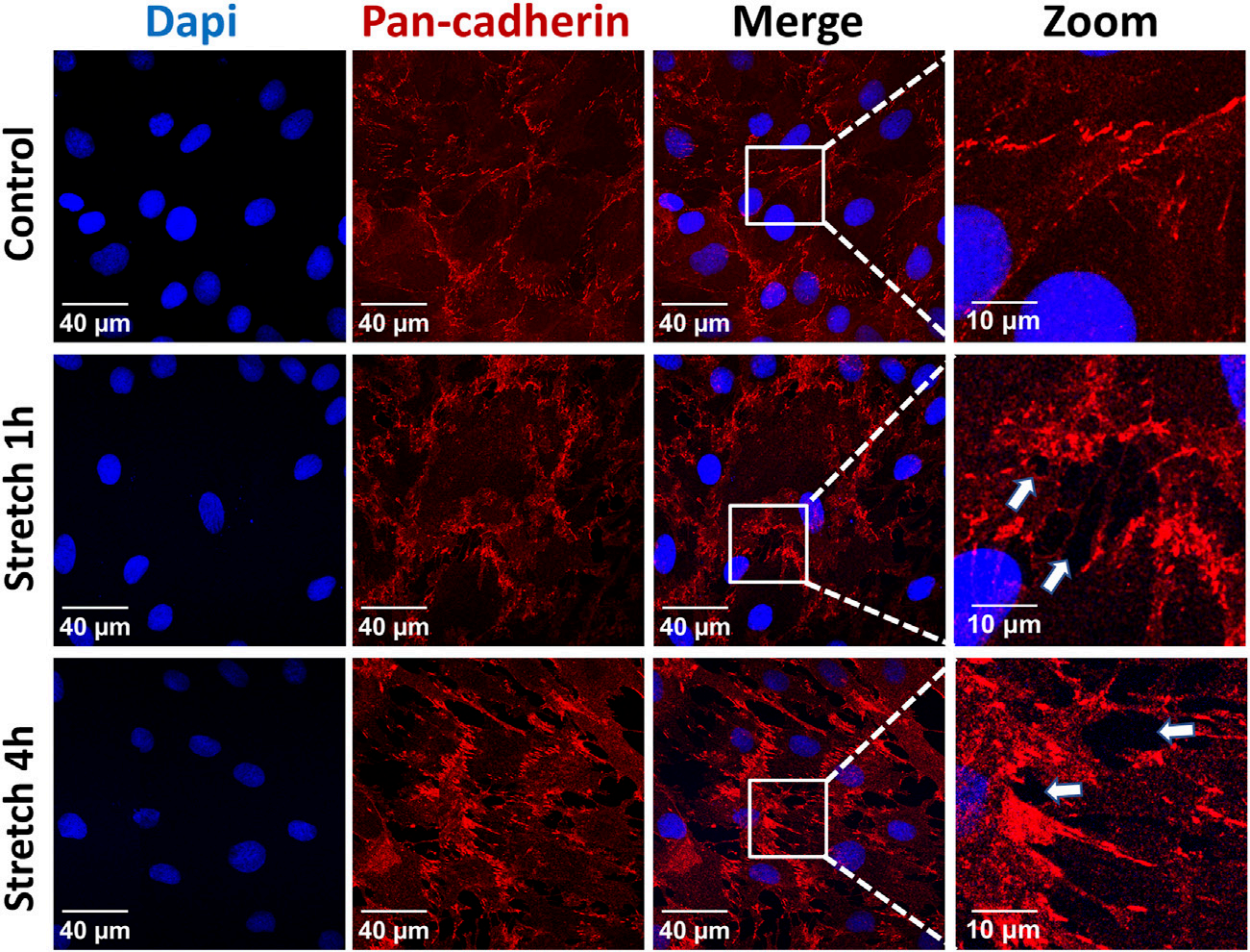
Effects of 30% cyclic stretch on membrane structure in rat alveolar epithelial type II L2 cells. Cells were grown on silicone membranes and subjected to 30% cyclic stretch for 1–4 h, while cells kept under static conditions (non-stretched) served as control. Intercellular junctions were identified with pan-cadherin (red), and nuclei with DAPI (blue). Scale bar: 40 μm; 10 µm (zoom). They are marked by arrows (n = 6).

The identity of the type II alveolar epithelial cells was confirmed by immunostaining with the specific marker SP-C (Supplementary Figure S1), as previously described (Ferreira et al., 2022). Application of 30% cyclic stretch for 1 hour and 4 hour caused the remodeling of the cytoskeleton and membrane deformation of AEC II cells. Cyclic stretch (30%) resulted in changes in cell morphology and orientation of the cytoskeleton (rearrangement of actin bundles, paracellular gaps and disrupted intercellular contacts). Representative images of F-actin immunostaining (labelled in green) are shown in Figure 1. In addition, intercellular junctions and cell-cell contacts were impaired in cells exposed to 30% cyclic stretch, compared to non-stretched controls, as shown by pan-cadherin staining (Figure 2).

### 3.2 YAP was activated by cyclic stretch in L2 cells

**FIGURE 3:**
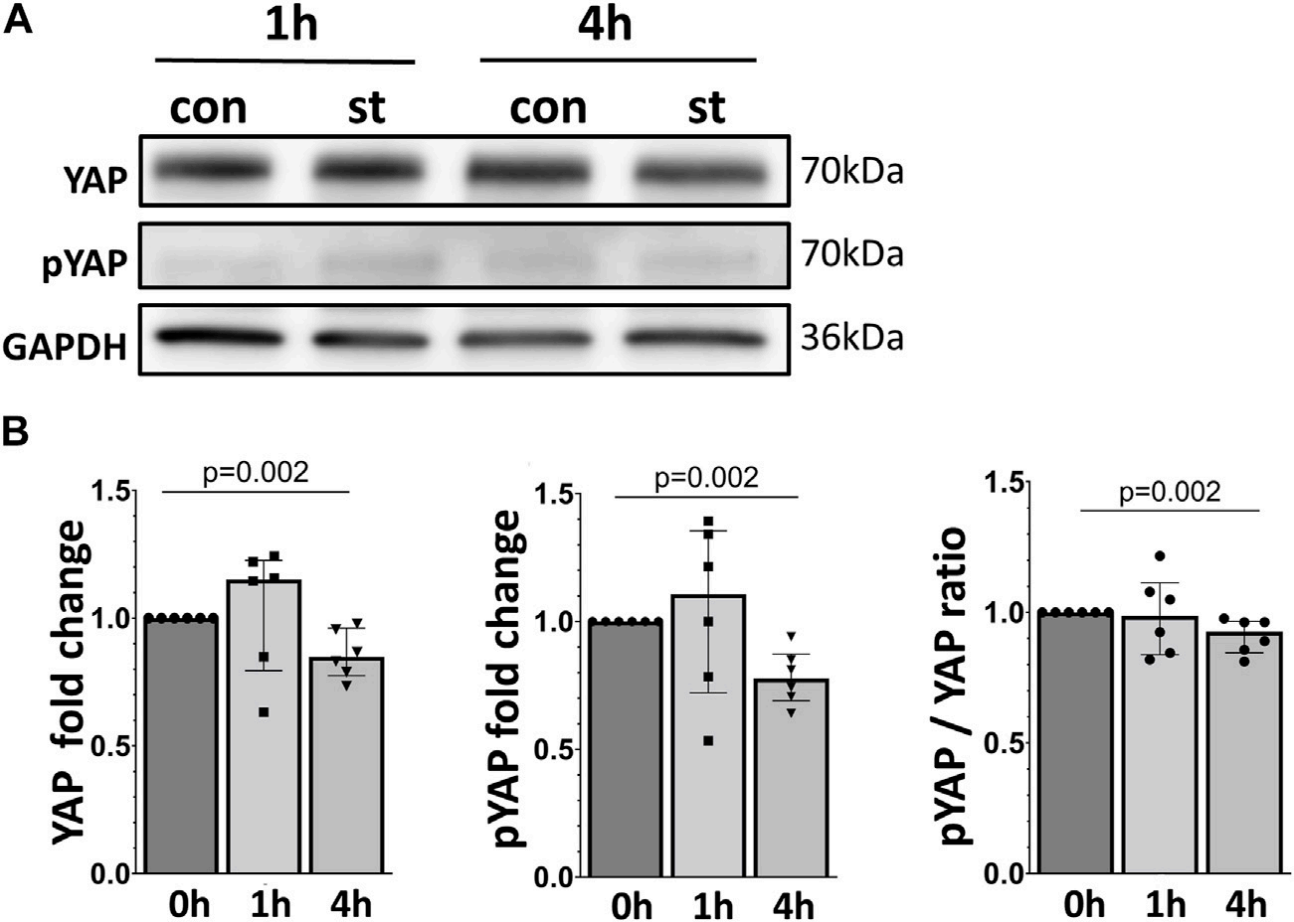
Effect of 30% cyclic stretch on total and phosphorylated YAP protein in AEC II. **(A)** Western blot analysis of whole cell lysates after 1 h and 4 h of 30% cyclic stretch, compared to non-stretched controls, using anti-YAP, anti-pYAP, and anti-GAPDH antibodies. For the full image, see Supplementary Figure S2. The bar graph in figure **(B)** shows relative levels of YAP and pYAP, normalized to GAPDH (as loading control), and the pYAP/YAP ratio. st: stretch. con: control. n = 6.

Protein expression of total (total YAP) and at Ser127 phosphorylated (p-YAP) YAP protein were analyzed by Western blot in total cell lysate (Figure 3). We did not detect a significant upregulation of total YAP or p-YAP after 1 h of 30% cyclic stretch, while both total YAP, p-YAP protein and p-YAP/YAP ratio decreased after 4 h of cyclic stretch compared to non-stretched control.

**FIGURE 4:**
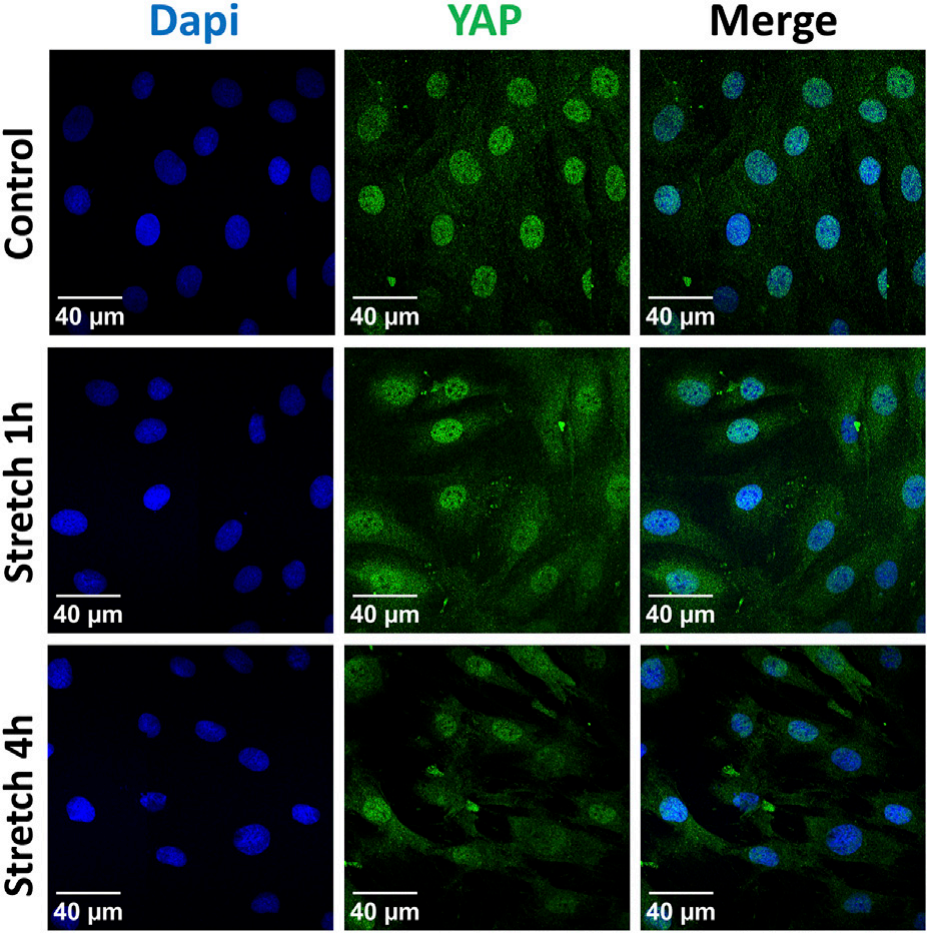
Localization of the YAP protein was analyzed by immunofluorescence after 1 h or 4 h of 30% cyclic stretch compared to non-stretched controls (static conditions). Confocal fluorescence microscope images show single channels for DAPI (DNA, blue) and YAP (green) and the merged image (right column). DAPI: 4′,6-diamidino-2-phenylindole. YAP: Yes-associated protein. Scale bar: 40 μm. n = 6.

Next, we analyzed the subcellular localization of YAP protein in response to 1 and 4 h of cyclic stretch was analyzed by immunofluorescence (Figure 4). Cells were labeled with a specific antibody against YAP and analyzed by immunofluorescence. After 1 h the ratio between nuclear and cytosolic YAP increased compared to the control cells.

**FIGURE 5:**
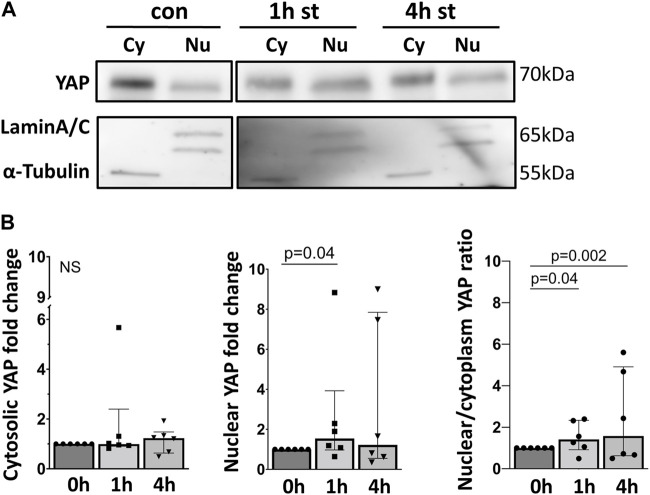
30% cyclic stretch increased nuclear YAP protein expression. **(A)** Western blot analysis of fractionated cell lysates of AEC II after 1 h and 4 h of 30% cyclic stretch, compared to non-stretched controls, using anti-YAP, anti-Lamin A/C (as nuclear protein loading control), and α-Tubulin (as cytosolic protein loading control). For the full image, see Supplementary Figure S3. The bar graph in Figure **(B)** shows the cytosolic and nuclear YAP protein expression (compared to static control) and the nuclear/cytosolic ratio after 1 and 4 h of 30% cyclic stretch. con: non-stretched control. st: stretch. Cy: cytosolic. Nu: nuclear. n = 6.

These findings were further confirmed, quantified by analyzing the YAP protein expression using Western blot in cytosolic and nuclear protein fractions of AEC II exposed to cyclic stretch (Figure 5). After 1 h of 30% cyclic stretch, nuclear YAP was significantly upregulated. We did not detect any statistical difference in nuclear YAP after 4 hours of 30% cyclic stretch. Furthermore, the nuclear/cytosolic YAP ratio was increased after 1 and 4 h compared to control.

### 3.3 Induction of YAP target genes Cyr61/CCN1 and CTGF/CCN2 by cyclic stretch in L2 cells

**FIGURE 6:**
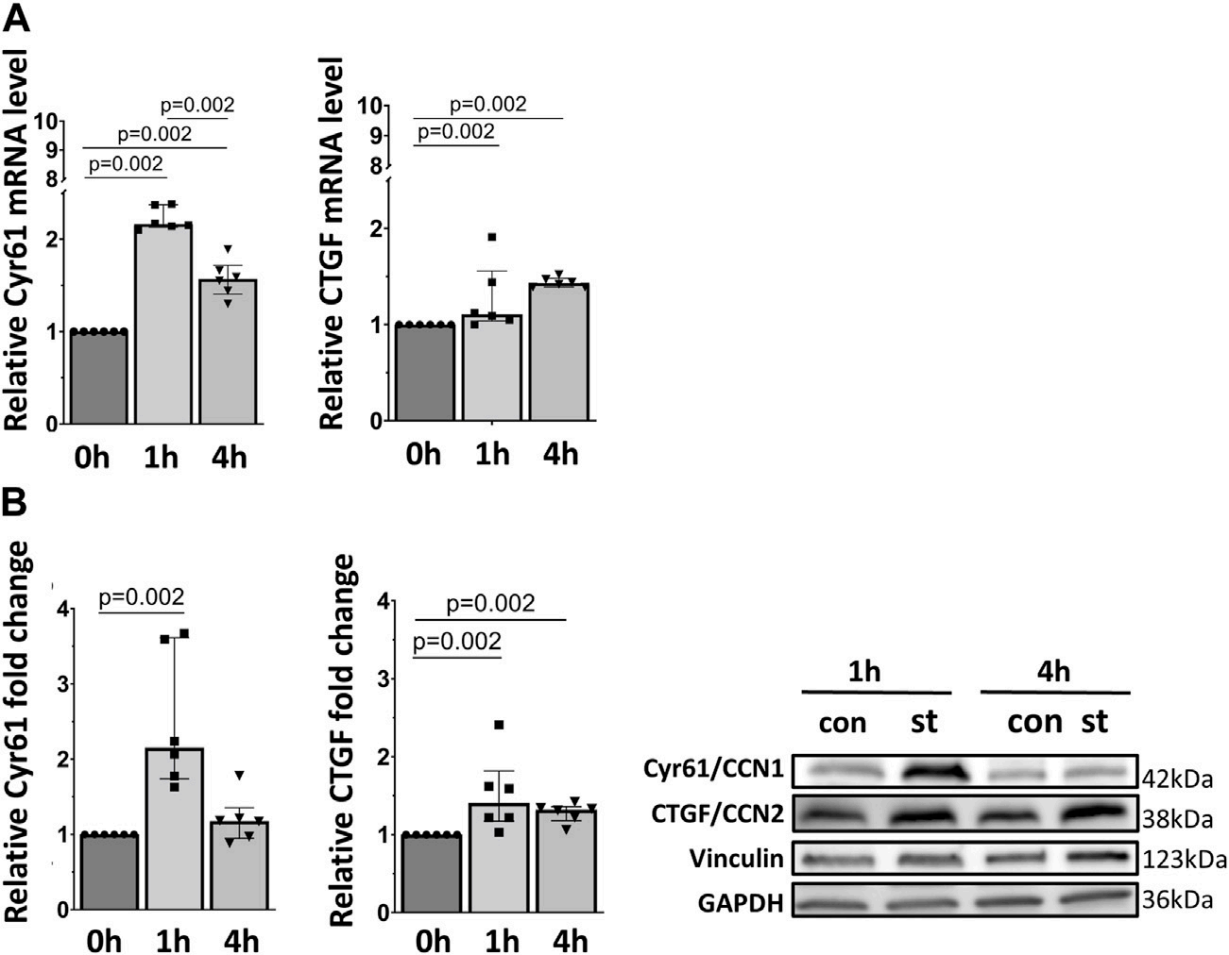
30% cyclic stretch upregulated YAP downstream target gene expression in AEC II. **(A)** The bar graphs show the Cyr61/CCN1 and CTGF/CCN2 mRNA expression by quantitative real-time PCR. The mRNA expression was normalized to β-Actin (as housekeeping gene) and non-stretched controls. **(B)** Western blot analysis of Cyr61/CCN1 and CTGF/CCN2 protein in whole cell lysates compared to non-stretched controls. For the full image, see Supplementary Figure S4. The bar graphs show relative levels of protein normalized to vinculin and GAPDH (as loading controls). con: control. st: stretch. n = 6.

Next, we analyzed the mRNA and protein expression of two YAP target genes, Cyr61/CCN1 and CTGF/CCN2. Both genes and proteins were increased by one and 4 h of cyclic stretch except for Cyr61/CCN1 protein in 1 h stretch (Figure 6).

### 3.4 30% cyclic stretch increases IL-6 mRNA and protein expression

**FIGURE 7:**
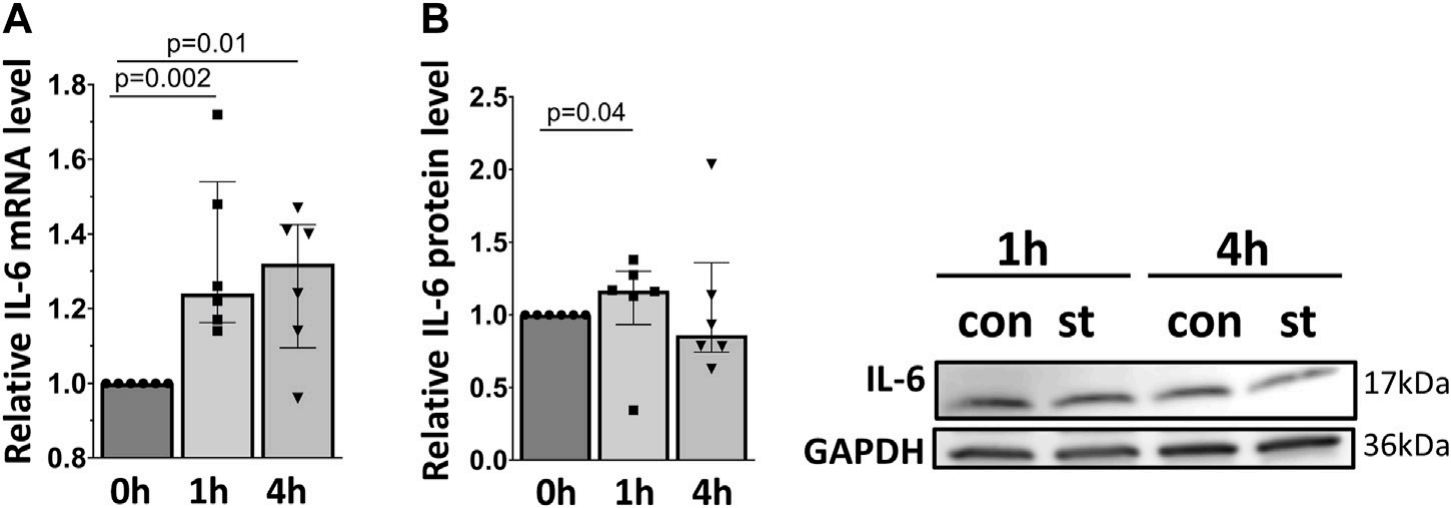
Effect of 30% cyclic stretch on IL-6 expression in rat alveolar epithelial Type II L2 cells. Cells were grown on silicone membranes and subjected to 30% cyclic stretch for 1 and 4 h. Cells kept under static conditions (non-stretched) served as the control. **(A)** The bar graph shows IL-6 mRNA expression by quantitative real-time PCR. The mRNA expression was normalized to β-Actin (as housekeeping gene) and non-stretched controls. **(B)** Western blot analysis of IL-6 protein expression in lysates of cells after 1 h and 4 h 30% stretch compared to non-stretched controls. For the full image, see Supplementary Figure S5. The bar graph shows the relative level of IL-6 protein normalized to GAPDH (as loading control). con: control. st: stretch. n = 6.

Figure 7 shows the quantitative RT-PCR and Western blot data of cellular IL-6 mRNA and protein expression in response to 30% cyclic stretch. Compared to static control, after 1 and 4 h of 30% cyclic stretch, IL-6 mRNA relative expression was upregulated (Figure 7A). The IL-6 protein expression was significantly induced after 1 h (Figure 7B) compared to the static control.

### 3.5 Regulatory role of YAP on IL-6 expression in cyclic stretched alveolar epithelial cells

**FIGURE 8:**
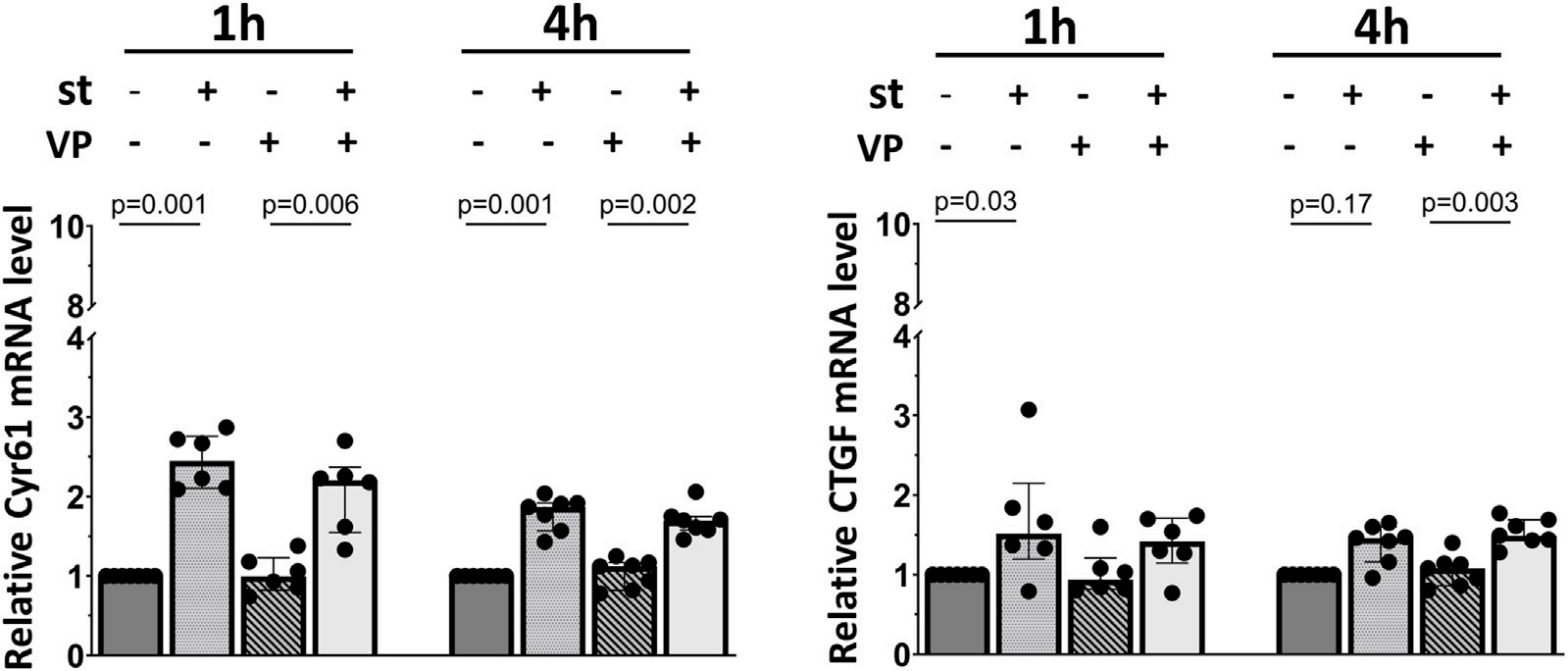
Influence of YAP inhibition on YAP downstream gene mRNA expression. L2 cells were pretreated with 2 μM of VP (YAP inhibitor) for 1 h prior to the exposure to 30% cyclic stretch for 1–4 h. The bar graphs show the Cyr61/CCN1 and CTGF/CCN2 mRNA expression by quantitative RT-PCR. The mRNA expression was normalized to β-Actin (as housekeeping gene) and compared to DMSO (as vehicle control). VP: verteporfin. st: stretch. n = 6–7.

Finally, we analyzed the impact of YAP inhibition on the expression of its target genes in response to cyclic stretch in alveolar epithelial cells (Figure 8). Cells were pretreated with the YAP inhibitor verteporfin (VP, 2 µM) prior to the exposure to cyclic stretch for 1 h. No changes were detected on the Cyr61/CCN1 and CTGF/CCN2 mRNA expression (Figure 8).

**FIGURE 9:**
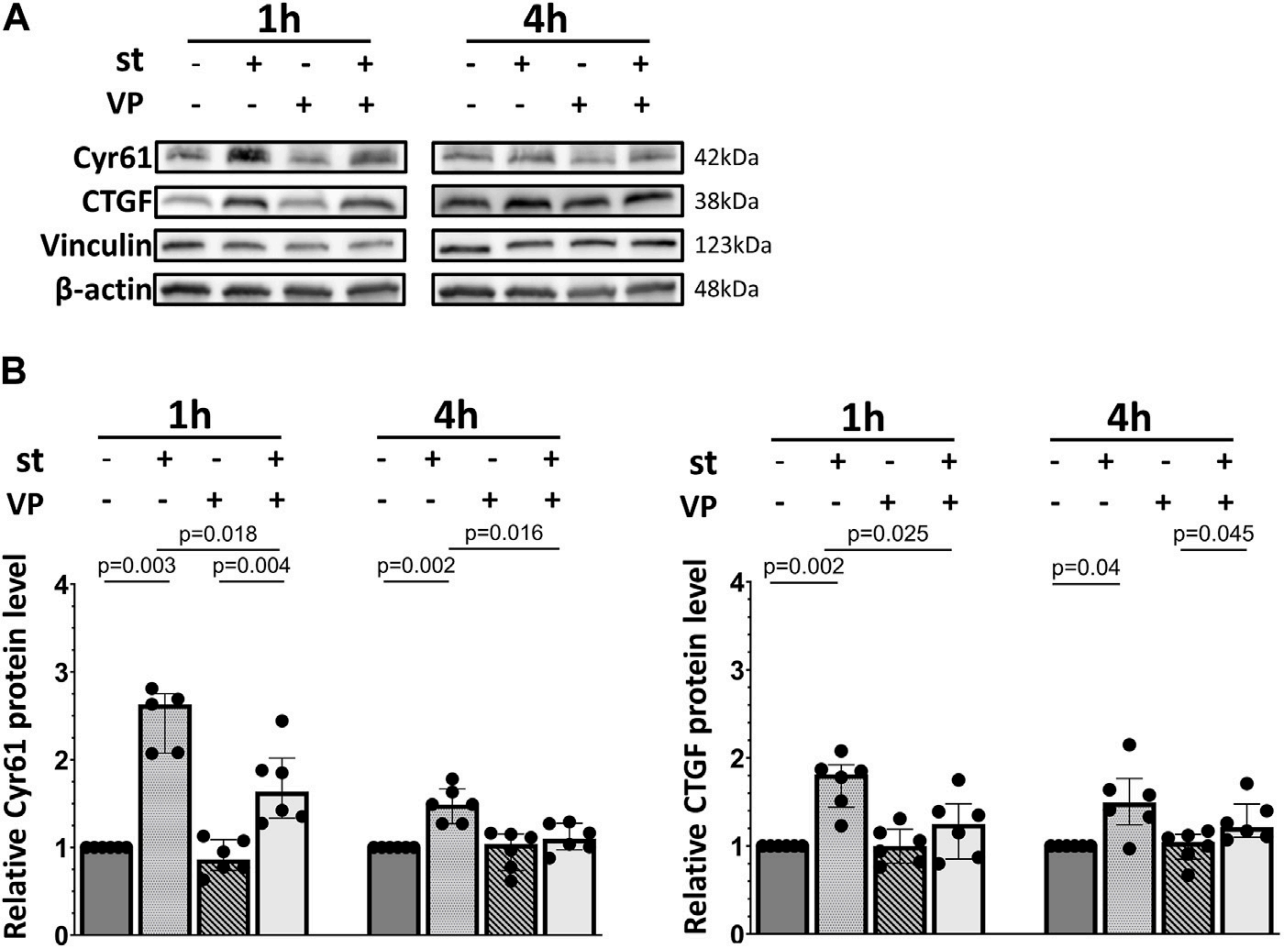
Influence of YAP inhibition on YAP downstream Cyr61/CCN1 and CTGF/CCN2 protein expression. L2 cells were pretreated with 2 μM of VP (YAP inhibitor) for 1 h prior to the exposure to 30% cyclic stretch for 1–4 h. **(A)** Western blot of cell lysates analyzed by anti-Cyr61/CCN1, CTGF/CCN2, vinculin, and β-Actin antibodies. For the full image, see Supplementary Figure S6. **(B)** Bar graphs show ratio of Cyr61/CCN1 and CTGF/CCN2 proteins normalized to loading controls. VP: verteporfin. st: stretch. n = 5-6.

In contrast, Cyr61/CCN1 protein upregulation was significantly blocked by YAP inhibition after 1 h of cyclic stretch (Figure 9). Furthermore, the CTGF/CCN2 protein upregulation was almost reduced to baseline levels (Figure 9). VP treatment without cyclic stretch (as control) did not affect the basal Cyr61/CCN1 and CTGF/CCN2 protein expression.

**FIGURE 10:**
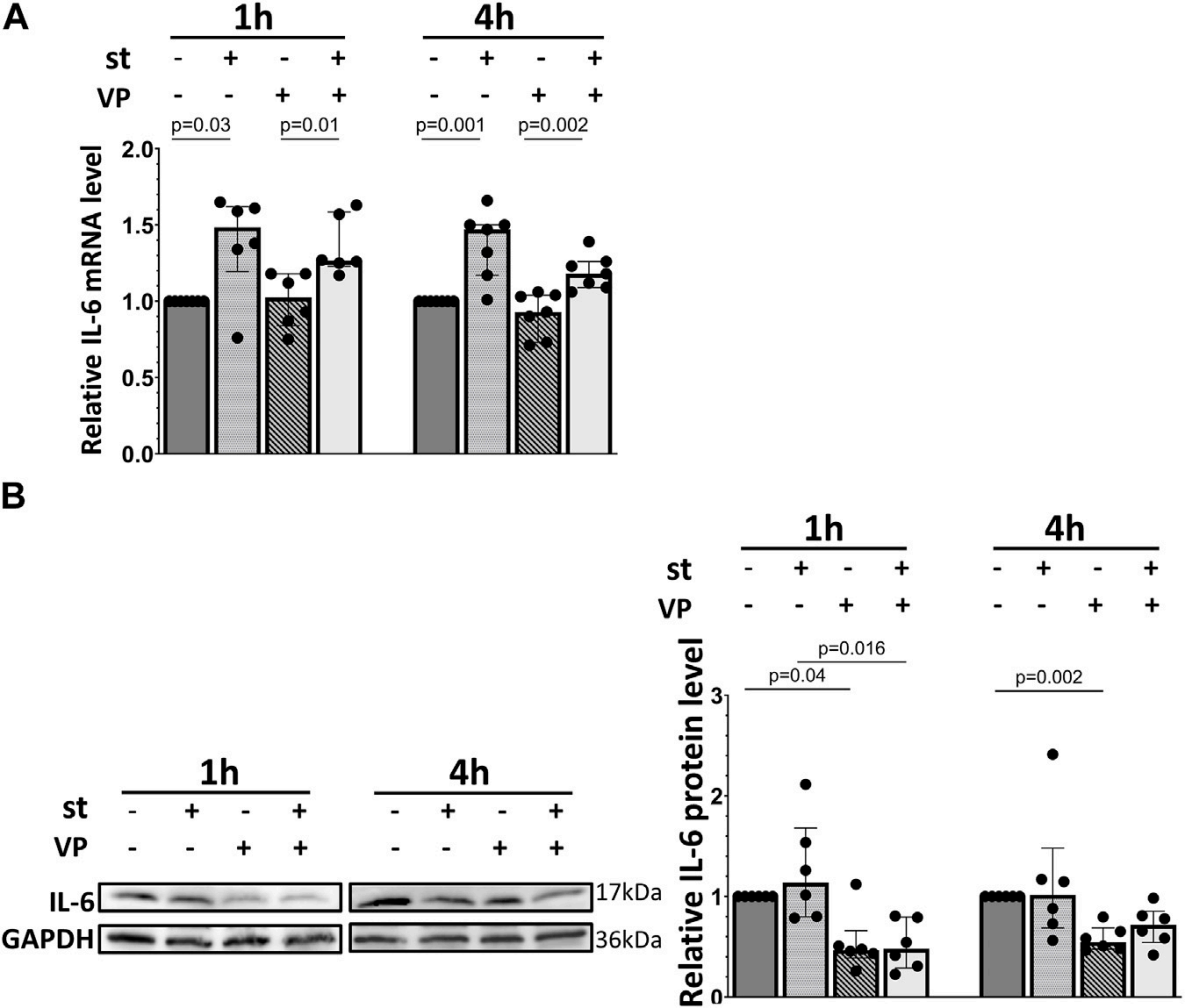
Impact of YAP inhibition on IL-6 expression of L2 cells in response to 30% cyclic stretch. L2 cells were pretreated with 2 μM of VP (YAP inhibitor) for 1 h prior to the exposure to 30% cyclic stretch for 1–4 h. **(A)** The bar graph shows the IL-6 mRNA expression by quantitative RT-PCR. The mRNA expression was normalized to β-Actin ratio (as housekeeping gene) and DMSO (as vehicle control). n = 6-7. **(B)** Western blot of cell lysates analyzed with anti- IL-6 and GAPDH antibodies. For the full image, see Supplementary Figure S7. The bar graph shows the IL-6 protein expression normalized to GAPDH (as loading control). n = 5-6.

Basal and stretch-induced IL-6 mRNA expression was not affected by VP pretreatment (Figure 10A). However, the IL-6 protein expression was downregulated by VP under static conditions and after 1 h of 30% cyclic stretch (Figure 10B).

## 4 Discussion

The main finding of the present study is that 1 hour of 30% cyclic stretch induces YAP nuclear accumulation and downstream target gene expression in alveolar epithelial cells, compared to the static control. Furthermore, YAP inhibitor verteporfin attenuates the YAP activation in response to 30% cyclic stretch and downregulates the IL-6 protein expression.

To our knowledge, this is the first study addressing the role of YAP on the inflammatory response to 30% cyclic stretch in alveolar epithelial cells *in vitro*. Our experiments were conducted in alveolar epithelial L2 cell line because it provides stable growth and exhibits typical type II cell characteristics, including the surfactant lamellar bodies (Rooney et al., 1975; Douglas et al., 1983; Liu et al., 2009). A major strength in this study is that our designed stretching device allows to mimic the mechanical stress patterns that AECs are submitted to *in vivo*, during MV, since there is a considerable body of evidence that mechano-transduction plays a key role in VILI (Chen et al., 2018). A cell-specific marker of type II pneumocytes is the surfactant protein C (SP-C). First, the identity of L2 cells was confirmed by SP-C immunofluorescence. Similar results were obtained in studies using human and rodent AEC II (Liang et al., 2016; Lemke et al., 2017; Tseng et al., 2021). Previous studies developed the application of 30% cyclic stretch as a model of pathological over-distension of alveolar epithelial cells during mechanical ventilation *in vivo* (Ferreira et al., 2022). The relevance of cell deformation as an *in vitro* model of excessive lung inflation has been reported in previous studies (Ferreira et al., 2022). When the lung volume increases by 40%–100% of the total lung capacity, a corresponding increase by 34%–35% of the basal surface area of alveolar epithelial cells has been suggested (Tschumperlin and Margulies, 1999; Tschumperlin et al., 2000).

Considering the current limitations of existing rodent models of mechanical ventilation to a time frame of a few hours and the rapid response of YAP expression and inflammation, our study was performed for one and 4 hours. Our data show that the over-distension stretch model is effective in inducing cell deformations in the intervention group. We noticed after 30% cyclic stretch a remodeling of the cytoskeleton including formation of F-actin stress fibers and an increased paracellular permeability. Similar changes in the cytoskeleton have been reported after stretching of epithelial and endothelial cells (Cavanaugh et al., 2006; DiPaolo et al., 2010; Davidovich et al., 2013; Huang and Helmke, 2015). In general, actin filaments are also involved in the sensing of increased physical forces and the intracellular mechanocoupling. Moreover, the F-actin and the cadherin may interact with the YAP protein during mechanosensing (Wennmann et al., 2014; Mohri et al., 2017). Therefore, we propose that cytoskeleton rearrangements during cellular deformation of over-stretched AEC II monolayers might be accompanied by intracellular YAP regulation.

To evaluate the potential activation of YAP by cyclic stretch in our model, we first examined its protein expression. We found after 1 hour of 30% cyclic stretch no significant change in the total YAP protein expression, but an increase in the nuclear YAP protein concentration. Our data are consistent with the hypothesis that cyclic stretch increases nuclear YAP accumulation. As a transcriptional coactivator, the YAP protein has been investigated in different biomechanical models using specific cell substrates, leading to F-actin accumulation and changes in cell shapes (Dupont et al., 2011; Fernández et al., 2011; Sansores-Garcia et al., 2011; Wada et al., 2011; Aragona et al., 2013). Next, we hypothesized that after translocation into the nucleus, YAP increases the transcription of specific targets. The upregulation of expression of YAP target genes Cyr61/CCN1 and CTGF/CCN2 supports this hypothesis. Total and phosphorylated YAP protein levels were reduced by 30% cyclic stretch after 4 h, while the nuclear/cytosolic ratio of YAP was still higher compared to the non-stretched controls. This finding indicates that 30% cyclic stretch may promote YAP activation by nuclear translocation without upregulation of total YAP protein. Exposure to less than 30% mechanical stretch led to nuclear translocation of YAP and modulation of YAP target genes in epithelial cells from other organs (Codelia et al., 2014; Strippoli et al., 2020). However, this pathway has not been investigated in alveolar AEC II so far. Furthermore, we detected that IL-6 mRNA expression and protein levels were upregulated by cyclic stress in alveolar cells. These data are supported by *in vitro* (Lionetti et al., 2005) and *vivo* studies (Tremblay et al., 2002) showing an increased expression of IL-6 during hyperinflation in isolated blood-free perfused mouse lungs (Uhlig et al., 2004).

Considering the potential crosstalk between YAP and innate immunity (Kapoor et al., 2011; Lv et al., 2018), we hypothesized that YAP activation might be involved in the stretch-induced IL-6 regulation in AEC II. This was evaluated using Verteporfin (VP), a well-characterized pharmaceutical inhibitor of the YAP axis. VP is a benzoporphyrin derivative and has been reported to inhibit the nuclear YAP-TEAD (TEA domain) interaction by intervening with YAP binding to TEADs’ surface and subsequently suppressing its transcriptional activity (Pobbati and Hong, 2020). Currently, VP has served as the primary pharmacological tool for investigating the role of YAP in various experimental models, and the dose of 2 µM is generally accepted (Wei and Li, 2020). Our data show that 2 µM VP treatment attenuates the YAP-driven expression of its target gene Cyr61/CCN1. It further reduced the CTGF/CCN2 target gene expression to basal levels. These findings support previous evidences that mechanical translation affects the activation of multiple signaling cascades, culminating in the reprogramming of gene expression and the production of growth factors such as Cyr61/CCN1 and CTGF/CCN2 by orchestrating complex aspects of cell trafficking and modulating cell signaling (Chaqour and Goppelt-Struebe, 2006). Other studies reported a downregulation of Cyr61/CCN1 and CTGF/CCN2 expression by 2 µM VP during longer treatment in gastric cancer and glioma cell lines (Al-Moujahed et al., 2017; Kang et al., 2018). A possible explanation for these differences could be the use of different cell types, and the longer duration of inhibition.

Interestingly, we found that the IL-6 protein level was reduced in both internal controls and VP treated samples, indicating that inhibition of YAP transcriptional activity may downregulate IL-6 protein independently of 30% cyclic stretch. Similar results of IL-6 induced by YAP were shown in cancer cells (Zhou et al., 2018; Wang et al., 2019), while other studies reported that suppression of YAP might prolong and worsen the inflammation in lung tissue (LaCanna et al., 2019; Li et al., 2022). A possible explanation for these different observations could be the crosstalk with other signaling pathways in YAP-IL-6 regulation. Mechanical forces can activate various mechanosensitive signaling pathways that can elevate IL-6 levels (Lionetti et al., 2005), including the Ras-Raf-Mitogen-activated protein kinases (MAPK) signaling axis (Correa-Meyer et al., 2002; Boopathy and Hong, 2019). In addition to that, our previous study (Rentzsch et al., 2017) showed the classical MAPK enzyme involved in cyclic stretching. Furthermore, MAPK signaling cascade is required for YAP activation in Mechanical-Tension-Induced Pulmonary Alveolar Regeneration (Liu et al., 2016). These pathways have the potential to modulate YAP activity and, in turn, may influence the expression of downstream target genes such as IL-6. Furthermore, VP may partly bypass YAP and target IL-6 directly or via other pathways, as the tumor-inhibitory effects of VP were reported to be YAP-independently (Zhang et al., 2015; Dasari et al., 2017). Further investigations might elucidate the interplay of YAP activation with other mechanosensitive signaling cascades and their role in IL-6 activation in biaxially stretched AEC II monolayers.

### 4.1 Limitations

The present study has several limitations. First, *in vitro* conditions do not fully reproduce the complex environment of the lung parenchyma *in vivo*, such as the three-dimensional architecture, elasticity, and stiffness, as well as the presence of the surrounding substrate. Second, although our device was designed to apply an equibiaxial strain field, a fraction of cells in the well’s periphery were exposed to strains of less than 30% due to one side fixation at the edge. This introduces a minor uncertainty about the observed mechanical threshold and partial inhomogeneity. Third, although VP is undoubtedly the most popular YAP inhibitor within the scientific community, the molecular details, of how VP binds to YAP, are still poorly understood. As an example, the tumor-inhibitory effects of VP are reported to be YAP-independent (Zhang et al., 2015; Dasari et al., 2017). In these studies, the antiproliferative and cytotoxic effects of VP were YAP-independent in endometrial cancer cells.

### 4.2 Conclusion

In rat alveolar epithelial cells, 30% cyclic stretch activated the expression of YAP target genes Cyr61/CCN1, CTGF/CCN2, and proinflammatory IL-6, which was attenuated by a YAP inhibitor. These findings indicate that YAP might be involved in the inflammatory alveolar epithelial response to ventilator-induced lung injury. Its inhibition might provide a novel therapeutic option for the treatment of ventilator-induced biotrauma.

## Data Availability

The original contributions presented in the study are included in the article/Supplementary Material, further inquiries can be directed to the corresponding author.
