# Supplementary material for: Modulation of the hippo-YAP pathway by cyclic stretch in rat type 2 alveolar epithelial cells—a proof-of-concept study
Source: Front Physiol. 2023 Oct 9;14:1253810. doi: 10.3389/fphys.2023.1253810 (PMC10591329; doi:10.3389/fphys.2023.1253810)
Supplement: Supplementary file 1 [file DataSheet1.docx]

Modulation of the Hippo-YAP Pathway by Cyclic Stretch in Rat Type 2 Alveolar Epithelial Cells – A Proof-of-Concept Study

Xi Ran^1, 2^, Sabine Müller^2^, Coy Brunssen^3^, Robert Huhle^2^, Martin Scharffenberg^2^, Christian Schnabel^4^, Thea Koch^2^, Marcelo Gama de Abreu ^2, 5, 6^, Henning Morawietz^3^, Jorge M C Ferreira^2^, Jakob Wittenstein^2^

^1^Department of Intensive Care Medicine, Chongqing General Hospital, China

^2^Department of Anesthesiology and Intensive Care Medicine, Pulmonary Engineering Group, University Hospital Carl Gustav Carus Dresden, TUD Dresden University of Technology, Dresden, Germany

^3^Division of Vascular Endothelium and Microcirculation, Department of Medicine III, University Hospital and Medical Faculty Carl Gustav Carus, TUD Dresden University of Technology, Dresden, Germany

^4^Department of Anesthesiology and Intensive Care Medicine, Clinical Sensoring and Monitoring Group, University Hospital Carl Gustav Carus Dresden, TUD Dresden University of Technology, Dresden, Germany

^5^Department of Intensive Care and Resuscitation, Anesthesiology Institute, Cleveland Clinic, Cleveland, OH, United States

^6^Department of Outcomes Research, Anesthesiology Institute, Cleveland Clinic, Cleveland, OH, United States

*** Correspondence:**Jakob Wittenstein, Department of Anesthesiology and Intensive Care Medicine, Pulmonary Engineering Group, University Hospital Carl Gustav Carus Dresden, TUD Dresden University of Technology, Dresden, Germany, Fetscherstr. 74, 01307 Dresden.
Jakob.wittenstein@ukdd.de

# Supplementary Figure


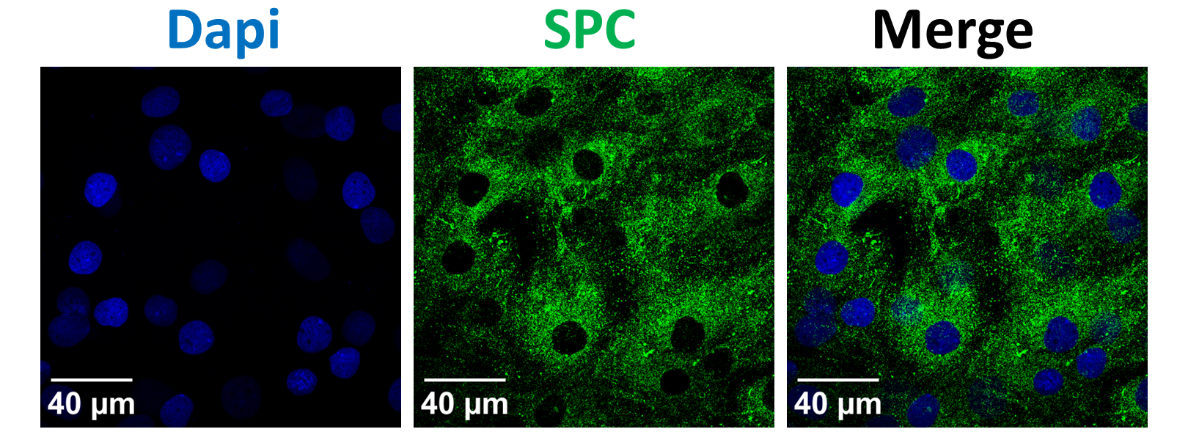


Supplementary figure 1 : Confirmation of identity of alveolar epithelial cell (AEC) type II line L2. Cells kept under static conditions (non-stretched) were stained with surfactant protein C (SP-C) as alveolar type II marker, and DAPI (as marker of DNA). Confocal fluorescence microscope images show single channels for DAPI (blue), SP-C (green) and the merged image. DAPI: 4′,6-diamidino-2-phenylindole.


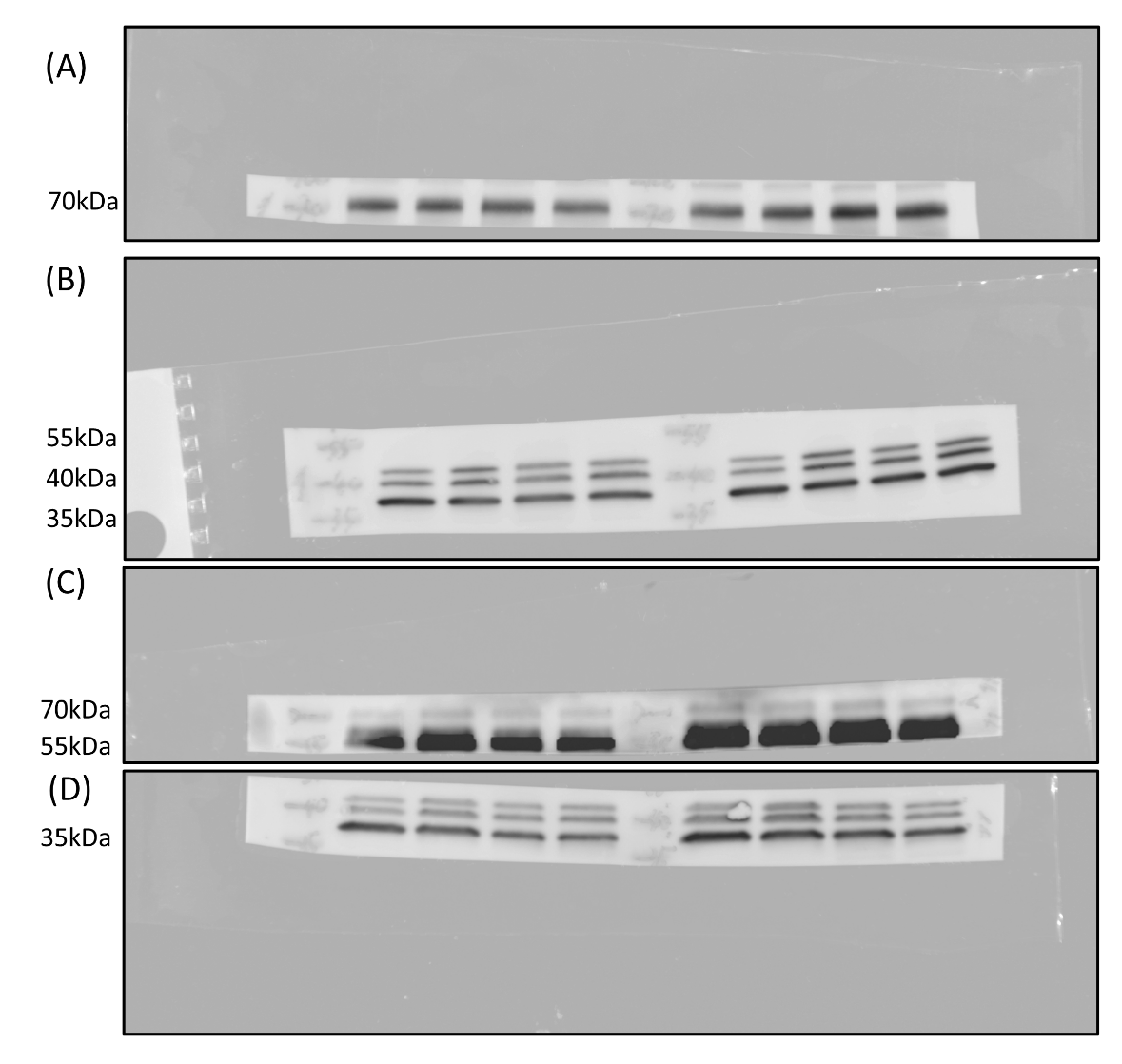


Supplementary figure 2 : Full image of Figure 3. Stripe A and B were cropped from the same blot. (A)Bands in Lanes 2 to 5 were cropped for the YAP image; (B) the lower thick bands in Lanes 2 to 5 of the same blot were cropped for the GAPDH image. Stripe C and D were cropped from the same blot. (C) The upper weak bands in Lanes 2 to 5 were cropped for the pYAP image; (D) the lower thick bands in Lanes 2 to 5 of the same blot were GAPDH (the loading control of pYAP).


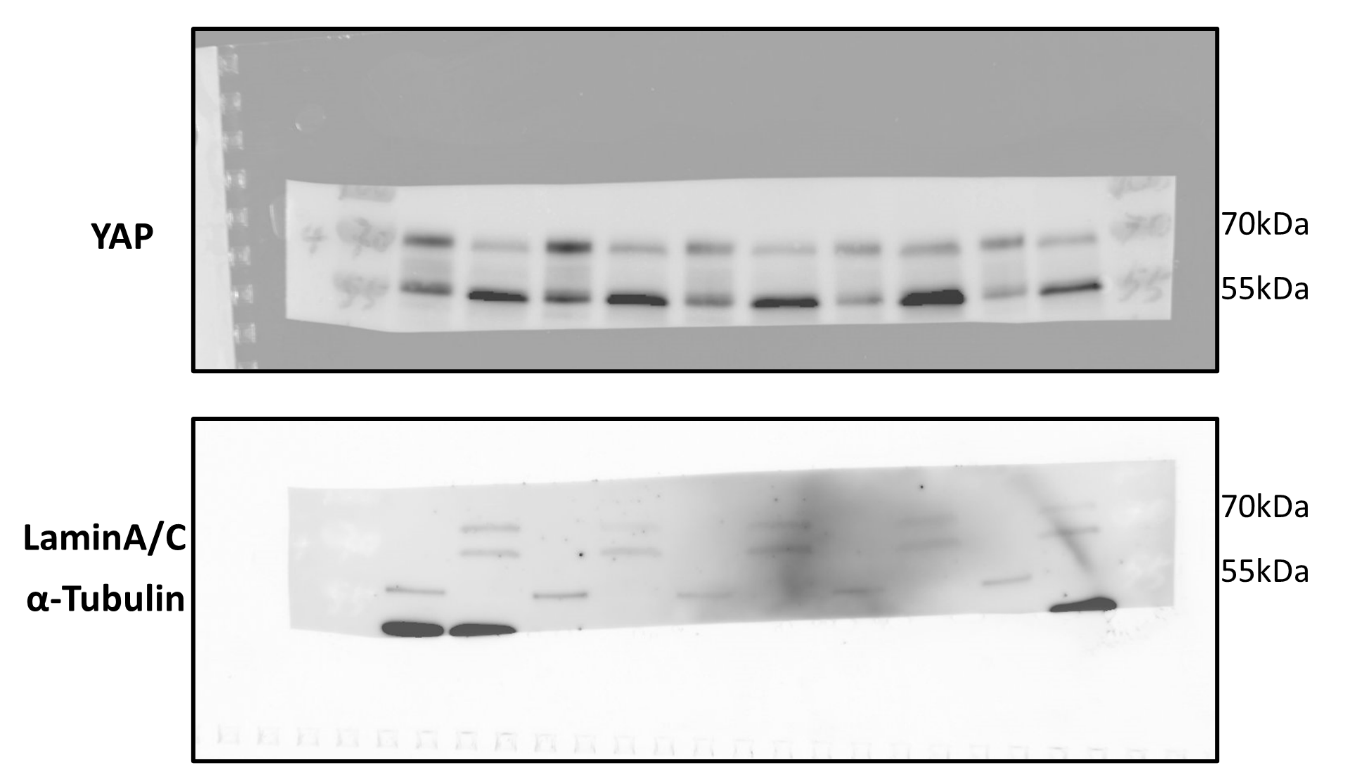


Supplementary figure 3: Full image of Figure 5. All antibodies were applied on the same stripe. Lanes 2 and 3 were cropped for control, and lanes 8 to 12 were cropped for one-hour and four-hours stretch conditions.


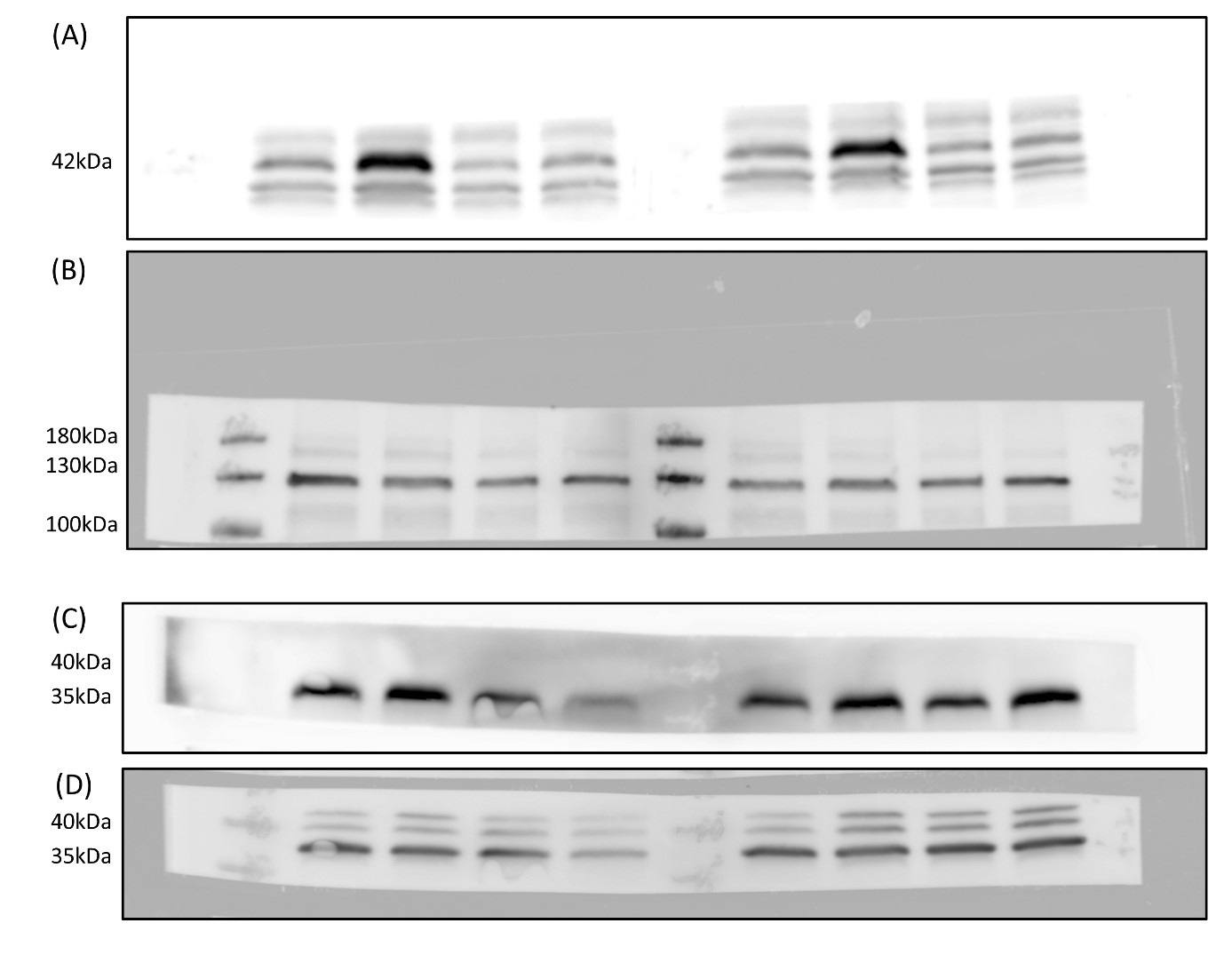


Supplementary figure 4 : Full image of Figure 6. Stripe A and B were cropped from the same blot. (A)The middle thick Bands in Lanes 7 to 10 were cropped for the Cyr61/CCN1 image; (B) bands in Lanes 7 to 10 of the same blot were cropped for the Vinculin image (the loading control). Stripes C and D are the same stripes. (C) Bands in Lanes 7 to 10 were cropped for the CTGF/CCN2 image; (D) the lower thick bands in Lanes 7 to 10 of the same stripe were GAPDH (the loading control).


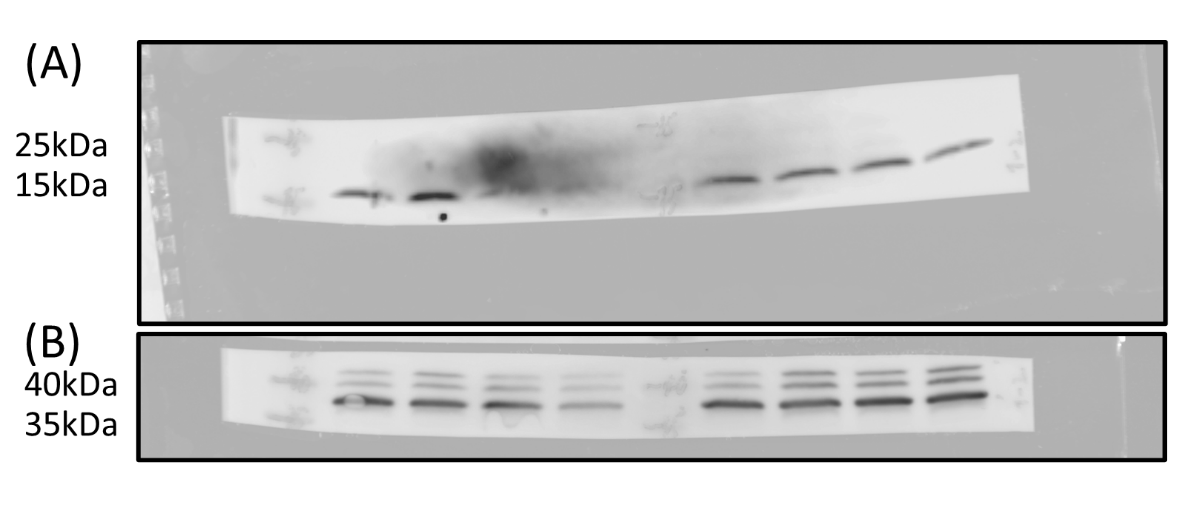


Supplementary figure 5 : Full image of Figure 7. Stripe A and B were cropped from the same blot. (A) Bands in Lanes 7 to 10 were cropped for the IL-6 image; (B)the lower thick bands in Lanes 7 to 10 of the same stripe were GAPDH (the loading control).


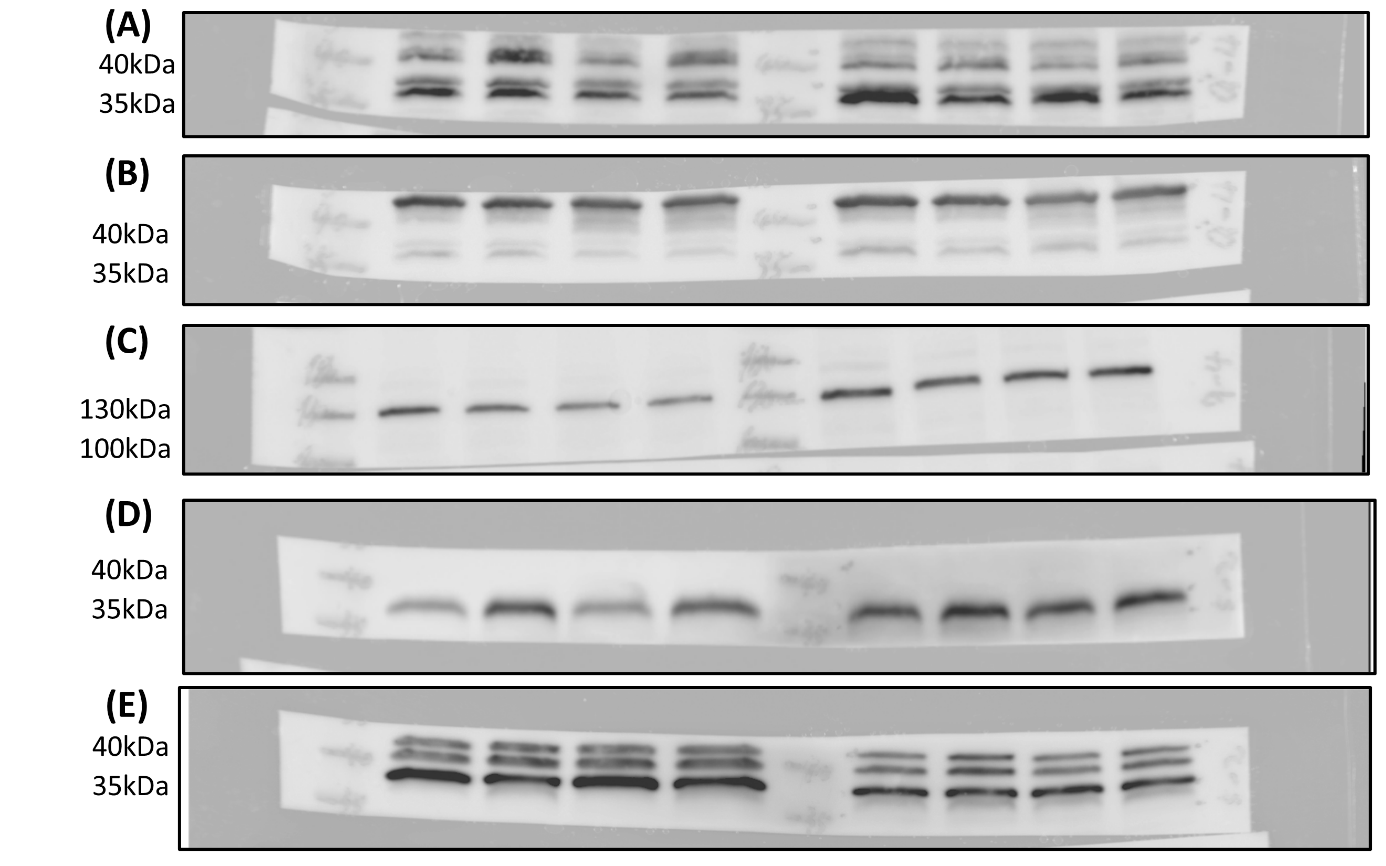


Supplementary figure 6 : Full image of Figure 9. Stripe A, B, and C were cropped from the same blot. Stripe A and B are the same stripe. (A)The upper thick Bands (near 42kDa) were cropped for the Cyr61/CCN1 image; (B) bands (near 48kDa) of the same stripe were cropped for the β-Actin (loading control of Cyr51/CCN1); (C) bands (near 123kDa) of the same blot were cropped for the Vinculin (loading control of Cyr51/CCN1). Stripes D and E are the same stripes. (D) Bands (near 38kDa) were cropped for the CTGF/CCN2 image; (E) the lower thick bands (near 36kDa) of the same stripe were cropped for the GAPDH(loading control of CTGF/CCN2).


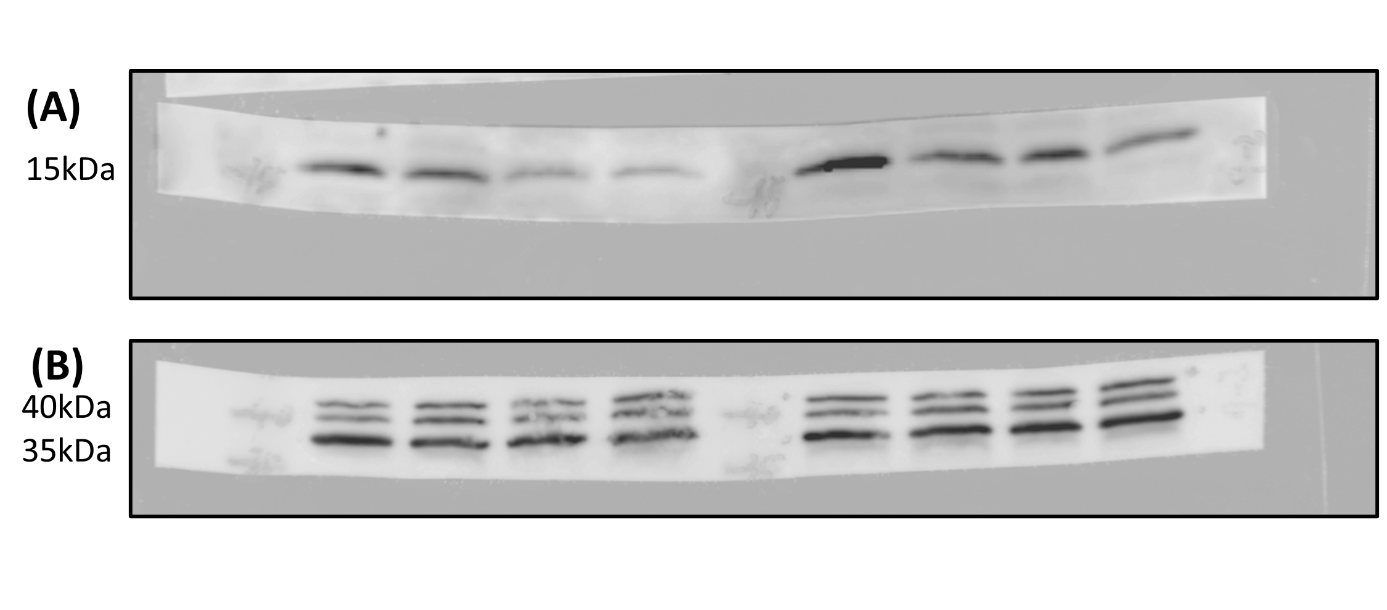


Supplementary figure 7: Full image of Figure 10. Stripe A and B were cropped from the same blot. (A) Bands were cropped for the IL-6 image; (B)the lower thick bands of the same stripe were GAPDH (the loading control).
